# Squamous cell carcinoma with sarcomatoid differentiation or carcinosarcoma of the uterine cervix associated with HPV33 infection: report of a rare case

**DOI:** 10.1186/s13000-020-00934-y

**Published:** 2020-02-08

**Authors:** Jan Hrudka, Blanka Rosová, Michael J. Halaška

**Affiliations:** 1 Department of Pathology, 3rd Faculty of Medicine, Charles University and Kralovske Vinohrady University Hospital, Ruská 87, 100 00, Praha 10 Prague, Czechia; 2 Department of Pathology and Molecular Medicine, 3rd Faculty of Medicine, Charles University and Thomayer Hospital, Prague, Czechia; 3 Department of Gynecology and Obstetrics, 3rd Faculty of Medicine, Charles University and Kralovske Vinohrady University Hospital, Prague, Czechia

**Keywords:** Sarcomatoid, Squamous carcinoma, Carcinsarcoma, Cervix, HPV

## Abstract

**Background:**

Squamous cell carcinoma is the most common malignant tumor of the uterine cervix with a well-documented link to infection with human papillomaviruses (HPV). According to a recent classification, there are several morphological variants of cervical squamous carcinoma, without reference to sarcomatoid squamous cell carcinoma, which is well described in other organs.

**Case presentation:**

In this paper, we describe an extremely rare case of a 77-year-old woman with primary malignant cervical tumor displaying biphasic histomorphology with an epithelioid and sarcomatoid part; the latter was immunohistochemistry positive for cytokeratin and vimentin. The association with a high-grade squamous intraepithelial lesion and molecular proof of HPV33 infection in the tumor tissue supported our diagnosis of carcinoma with partial sarcomatoid differentiation.

**Conclusion:**

We report a rare case of a primary cervical epithelial tumor with a partial sarcomatoid phenotype, an unequivocal HPV infection, and an associated precancerous lesion in the cervical mucosa. This is the first description of an HPV33 infection underlying a biphasic epithelioid-sarcomatous tumor of the uterine cervix. The terminology overlap between sarcomatoid carcinoma and carcinosarcoma is also discussed.

## Background

Squamous cell carcinoma (SCC) is the most common malignant tumor of the uterine cervix, whereas cervical cancer is the second or third most common malignancy in women worldwide [1]. The etiopathogenetic link to infection with human papillomavirus (HPV) and precursor squamous intraepithelial lesions in most cervical carcinomas are well known. The recent World Health Organization (WHO) Classification of gynecological tumors or Blaustein’s Monography [2] discerns several histomorphological variants of cervical SCC: keratinizing, non-keratinizing, basaloid, verrucous, warty/condylomatous, papillary, squamotransitional, and lymphoepithelioma-like carcinoma. However, the WHO Classification does not describe the rare sarcomatoid squamous cell carcinoma (SSCC) even though it is described in the literature [3–11]. In this paper, we report on the case of a rare SSCC of the uterine cervix with molecular proof of HPV33 infection.

## Case presentation

**Fig. 1:**
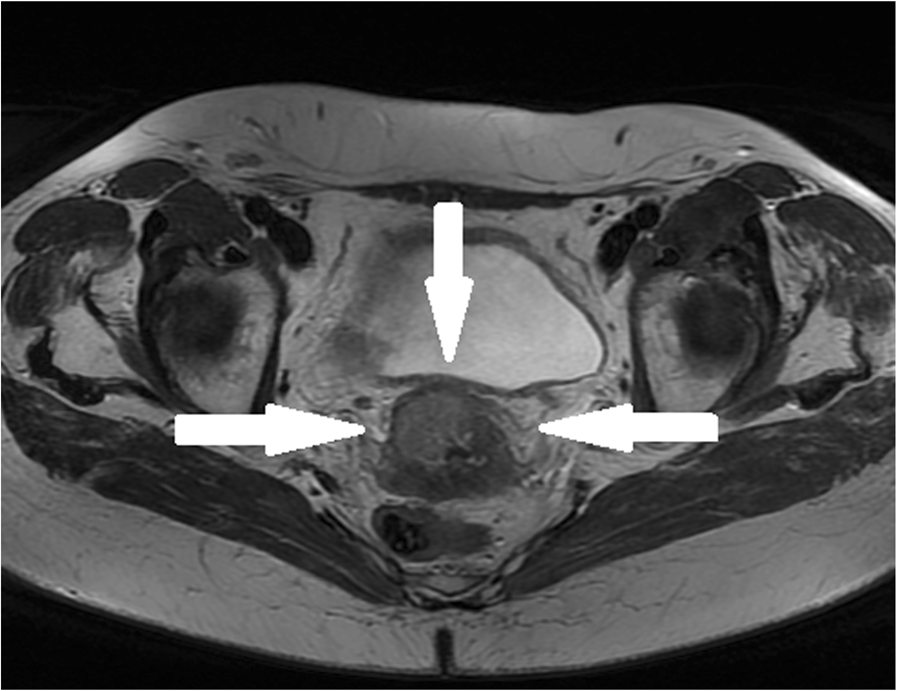
Magnetic resonance imaging showing a polypous tumor in anterior labium region of the cervix, with the size of 26x24x23mm, without parametrial infiltration, without lymphadenopathy

A 77-year-old Caucasian woman presented to the university hospital with vaginal bleeding that was occurring 30 years after menopause, and 45 years after her last gynecological examination. Ultrasonography and magnetic resonance imaging of the pelvis revealed a hypoechogenic well-circumscribed endophytic tumor measuring 30 × 28 × 24 mm, almost filling the entire bulk of the anterior cervical labium (Fig.1). A biopsy excision from the tumor mass was performed. Microscopically, it was a neoplastic tissue with a solid architecture consisting of polymorphous tumor cells containing giant partly lobulated, multiple nuclei, and prominent eosinophilic nucleoli. Immunohistochemistry was positive for pankeratin (cytokeratin) AE1/AE3, vimentin, and p16. These findings lead to a diagnosis of sarcomatoid carcinoma. The patient underwent radical hysterectomy and adnexectomy (Wertheim-Meigs surgery) with pelvic lymphadenectomy, together with identification and frozen histological examinations of the sentinel lymph nodes.

**Fig. 2:**
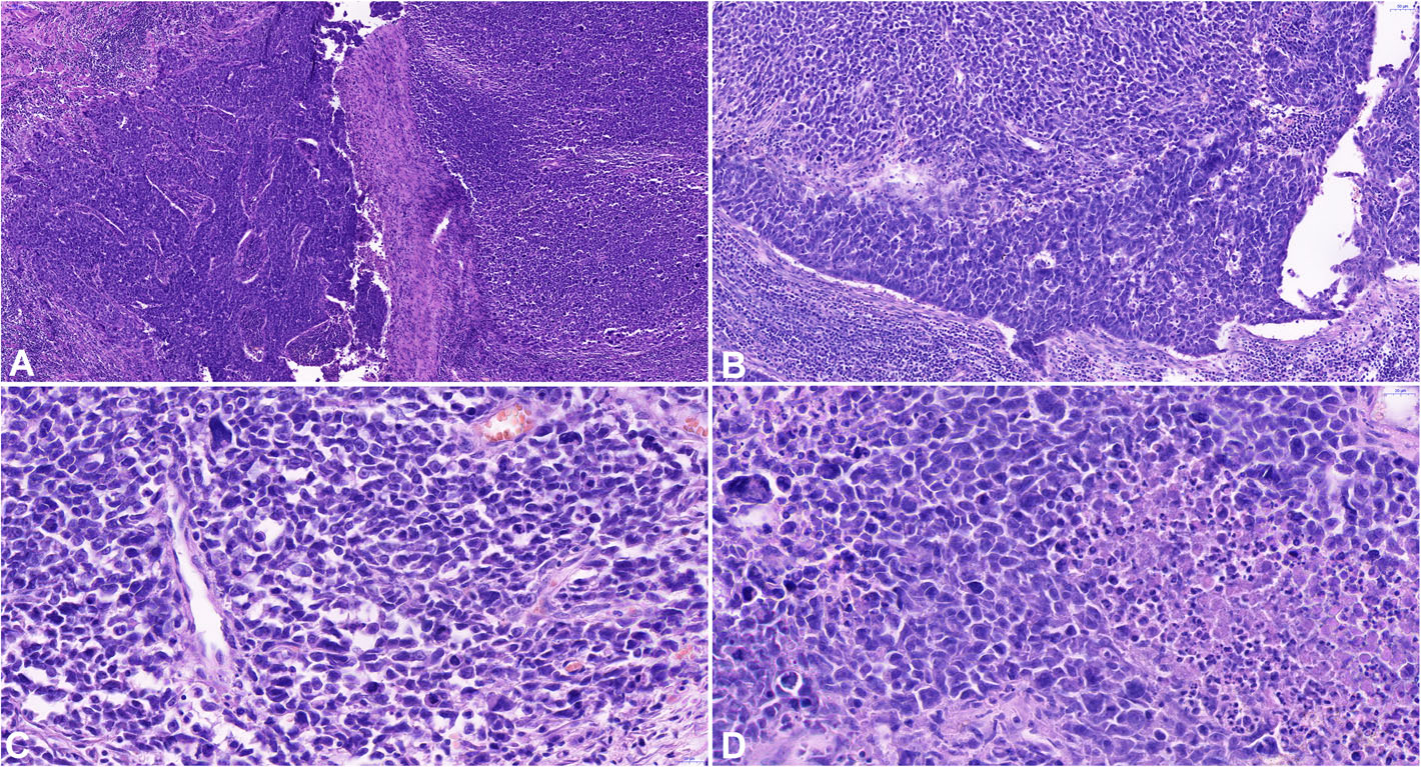
Scans of histological slides stained with hematoxylin-eosin, showing biphasic malignant tumor of the uterine cervix, **a** squamous epithelioid part in the left, sarcomatoid part in the right, 7,1x **b** direct transition between sarcomatoid (up) and epithelioid (bottom) tumor components, 18,2x **c** detail of the sarcomatoid part with highly atypical spindle cells and bizarre cells, 52,7x, **d** and with necrosis, 53,2x

**Fig. 3:**
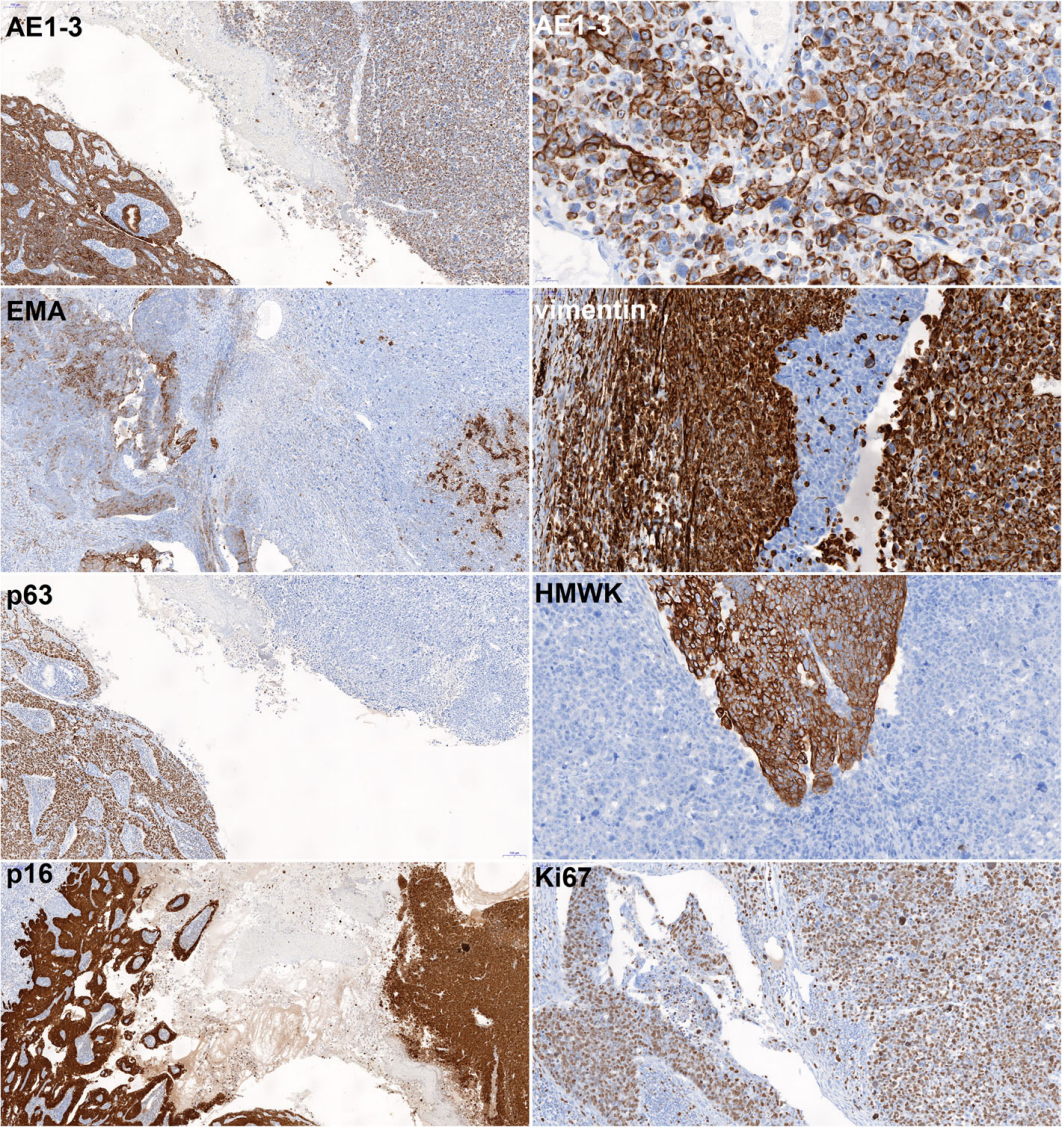
Scans of histological slides stained immunohistochemically. AE1/3 (11,2x): note the strong diffuse positivity in the epithelioid part of tumor (left) and weaker, but unequivocal diffuse positivity in the sarcomatoid part, in the upper right corner in detail (5,9x). EMA (7,8x): Note the heterogenous positivity in both epithelioid (left) and sarcomatoid (right) parts of the tumor. Vimentin (29,2x): note the strong positivity in the cervical stroma (left) and in the sarcomatoid part (right) and negativity in the accompanying HSIL (in the middle). P63 (11,2x): note the diffuse nuclear positivity in the epithelioid part (left) and negativity in the sarcomatoid tumor (right). HMWK (26,5x): note the positivity in the epithelioid tumor bud (in the middle) and full negativity in the sarcomatoid part. P16 (7,1x) note the strong and diffuse positivity in both parts of the tumor. Ki67 (16,6x): note the brisk proliferation activity in approximately 80% of tumor cells in both parts

The hysterectomy and lymphadenectomy specimens were sent for histopathological examination. Microscopically, an obviously malignant biphasic tumor, containing an epithelioid part with the morphology of invasive squamous non-keratinizing carcinoma and a polymorphous cell-rich component with abundant highly polymorphous, monstrous cells, with bizarre nuclear atypia and pleomorphism were observed (Fig.2). Immunohistochemistry (Fig. 3) was strongly positive for pankeratin (cytokeratin) AE1/AE3 in the epithelioid-squamous part and weaker but still unequivocally positive in the polymorphous part. Epithelial membrane antigen (EMA) was focally positive in both parts of the tumor. P63 and high-molecular-weight cytokeratin (HMWK) were positive in the epithelioid-squamous component, while the polymorphous component was negative. The entire tumor showed strong diffuse p16 positivity. The polymorphous component was vimentin positive while the epithelioid-squamous part was vimentin negative. Proliferation activity (Ki67) was present in approximately 80% of the tumor cell nuclei. The expression of p53 was wild type. The rest of the markers tested, i.e., estrogenic and progesterone receptor, Wilms tumor-1 (WT-1), smooth muscle actin, desmin, myogenin, CD56, and ERG, were negative. Based on squamous morphology and p63 positivity, together with the spindle cell polymorphous component, which was cytokeratin positive, the diagnosis of SCC with sarcomatoid differentiation was confirmed. The tumor measured 27 × 24 × 24 mm. There was no involvement of the parametrium and no lymphatic or vascular invasion. Surgical resection margins were tumor-free. All 17 of the lymph nodes found in the lymphadenectomy specimen were free of metastatic involvement, including 2 sentinel lymph nodes. The sizable extent of the high-grade squamous intraepithelial lesion/cervical intraepithelial neoplasia grade 3 (HSIL/CIN3), affecting almost the entire exocervix and involving the exocervical resection margins, was an interesting ancillary finding. Immunohistochemistry on the HSIL showed strong diffuse p16 positivity.

Using 2% agarose gel electrophoresis, we performed an additional polymerase chain reaction (PCR) of the HPV genes, using the deoxyribonucleic acid (DNA) extracted from the tumor tissue, using primers amplifying the E6/E7 gene region and searching for HPV types 16, 18, 31, 33, 35, and 45. The PCR reaction found HPV type 33 in the extracted DNA.

## Discussion

SSCC is a well-described variant of SCC in the lungs [3], the upper aerodigestive tract, and the skin, but rare in the uterine cervix [4]. SSCC is a primarily epithelial tumor composed of a squamous cell carcinoma element and a polymorphous sarcomatoid element derived from the squamous cell carcinoma element [3]. However, SSCC is not described in the recent WHO Classification of gynecological tumors. Kumar et al. (2008) reviewed only 16 cases [5]. From our point of view, the term SSCC overlaps to a significant extent with carcinosarcoma or malignant mixed Müllerian tumor (MMMT) of the uterine cervix, which is described in WHO as a very rare malignancy. There are more than 80 published cases of MMMTs of the uterine cervix [12]. Grayson et al. (2001) examined eight cases of uterine cervical carcinosarcomas (or MMMTs) and found an HPV-infection in all cases; in seven cases, it was found along with a squamous intraepithelial lesion (SIL) in the adjacent cervical epithelium [13], as in our case. Moreover, the authors documented cytokeratin-positivity in all eight tumors and EMA-positivity in the majority of the described cases, which was also similar to our case. Unlike Grayson et al., who found HPV type 16 in all cases, we found HPV type 33 in the DNA extracted from the tumor tissue. However, an infection with HPV16 was described in a single case of cervical SSCC as well [11].

Contrary to rare cervical MMMT or carcinosarcoma, MMMT of the uterine corpus is more common and has similar molecular and epidemiological characteristics to endometrial carcinoma, i.e., obesity and menopause [14], whereas uterine cervical MMMT and SSCC are HPV-related [11, 13]. Cervical MMMT differs in several ways from its much more common counterpart in the uterine corpus: the most common carcinomatous pattern in cervical MMMTs is a basaloid pattern that consists of anastomosing densely cellular trabeculae composed of small cells with scant cytoplasm and peripheral palisades; other epithelial patterns include typical squamous cell carcinoma. The sarcomatous element in MMMT is typically homologous and frequently has the appearance of a fibrosarcoma or endometrial stromal sarcoma [15]. Cervical MMMTs, compared to their counterparts in the corpus, are more commonly confined to the uterus at presentation, have a non-glandular epithelial component, and potentially have a better prognosis, whereas carcinosarcoma of the uterine corpus almost always has adenocarcinomatous component [16].

All considerations mentioned above lead us to the opinion that tumors of the uterine cervix with a concomitant epithelial and sarcomatous component are usually HPV-related and differ epidemiologically and histopathologically from corporal MMMT, independently of labeling them carcinosarcoma or sarcomatoid carcinoma. Due to the lack of a large dataset, it remains unclear, if the distinction between SSCC and cervical MMMT would have a clinical impact. According to the literature, cervical SSCC is quite an aggressive tumor [8]. From our point of view, the terminology overlap between cervical MMMT and SSCC may explain the paucity of literature surrounding SSCC. From our point of view, due to the unequivocal link with HPV infection, the term SSCC or SCC with sarcomatoid differentiation fits better than MMMT for this particular tumor; because of obviously squamous epithelial part of the tumor and well known relation between HPV and cervical SCC.

There are several theories about the potential histogenesis of biphasic malignant tumors of the female genital tract, including the “collision,” “combination,” “composition,” and “metaplastic” theories [13], which may be simplified to biclonal or monoclonal. Genetic studies [17–19] support the monoclonal origin of MMMTs from a common stem cell. Carcinosarcoma or sarcomatoid carcinoma may be regarded as an epithelial tumor originally in which a subclonal population has undergone a sarcomatoid transformation (or metaplasia): the common cytokeratin- and EMA-positivity of both the epithelial and sarcomatoid parts in our cases and other published cases may support this statement. The differential diagnosis between carcinosarcoma and sarcomatoid carcinoma remains rather an academic question; however, in the absence of definitive criteria, it’s etiological and morphological similarity to squamous carcinoma of the cervix leads us to prefer the term sarcomatoid carcinoma.

In conclusion, we report on an extremely rare case of primary cervical SSCC or carcinosarcoma (International Federation of Gynecology and Obstetrics (FIGO) stage 1B1 associated with HPV 33 infection). To the best of our knowledge, this is the first demonstration of HPV 33 in this type of tumor. The patient underwent adjuvant brachyradiotherapy, and five months after surgery, she is disease-free.

## Data Availability

It is not possible to share research data publicly.

## Abbreviations

CD: Cluster of differentiation
DNA: Deoxyribonucleic acid
EMA: Epithelial membrane antigen
FIGO: The International Federation of Gynecology and Obstetrics
HMWK: High-molecular-weight keratin
HPV: Human papillomavirus
HSIL/CIN3: High-grade squamous intraepithelial lesion/cervical intraepithelial neoplasia grade 3
MMMT: Malignant mixed Müllerian tumor
PCR: Polymerase chain reaction
SCC: Squamous cell carcinoma
SIL: Squamous intraepithelial lesion
SSCC: Sarcomatoid squamous cell carcinoma
WHO: World Health Organization
WT-1: Wilms tumor-1
